# Field investigation with real-time virus genetic characterisation support of a cluster of Ebola virus disease cases in Dubréka, Guinea, April to June 2015

**DOI:** 10.2807/1560-7917.ES.2018.23.12.17-00140

**Published:** 2018-03-22

**Authors:** Alessandro Pini, Delayo Zomahoun, Sophie Duraffour, Tarik Derrough, Myrna Charles, Joshua Quick, Nick Loman, Lauren Cowley, Mamadou Leno, Nobila Ouedraogo, Oumou Thiam, Alfonso Hernández-Romieu, Annie Iko, Halimatou Keita, Djiba Konate, Aboubacar Aboubak Soumah, Etran Bouchouar, Samuel Ileka-Priouzeau, Sakoba Keita, Boubacar Diallo, Fode Cisse, Josep Jansa, Miles Carroll, Stephan Günther, Ettore Severi, Pierre Formenty

**Affiliations:** 1 European Centre for Disease Prevention and Control (ECDC), Solna, Sweden; 2 The Public Health Agency of Sweden, Solna, Sweden; 3 Centers for Disease Control and Prevention, Atlanta, Georgia, United States; 4 Bernhard-Nocht-Institute for Tropical Medicine, Hamburg, Germany; 5 The European Mobile Laboratory Consortium, Bernhard-Nocht-Institute for Tropical Medicine, Hamburg, Germany; 6 Institute of Microbiology and Infection, University of Birmingham, Birmingham, United Kingdom; 7 Gastrointestinal Bacterial Reference Unit, Colindale, Public Health England, London, United Kingdom; 8 Direction Prefectural de la Santé, Dubréka, Guinea; 9 Postgraduate Training for Applied Epidemiology, Robert Koch Institute, Berlin, Germany; 10 World Health Organization, Conakry, Guinea; 11 Ministère de la Santé, Conakry, Guinea; 12 National Infection Service, Public Health England, Porton Down, Wiltshire, United Kingdom; 13 University of Southampton, South General Hospital, Southampton, United Kingdom; 14 World Health Organization, Geneva, Switzerland

**Keywords:** Ebola virus, laboratory surveillance, outbreaks, Guinea, viral haemorrhagic fever

## Abstract

On 11 May 2015, the Dubréka prefecture, Guinea, reported nine laboratory-confirmed cases of Ebola virus disease (EVD). None could be epidemiologically linked to cases previously reported in the prefecture. We describe the epidemiological and molecular investigations of this event. We used the Dubréka EVD registers and the Ebola treatment centre’s (ETC) records to characterise chains of transmission. Real-time field Ebola virus sequencing was employed to support epidemiological results. An epidemiological cluster of 32 cases was found, of which 27 were laboratory confirmed, 24 were isolated and 20 died. Real-time viral sequencing on 12 cases demonstrated SL3 lineage viruses with sequences differing by one to three nt inside a single phylogenetic cluster. For isolated cases, the average time between symptom onset and ETC referral was 2.8 days (interquartile range (IQR): 1–4). The average time between sample collection and molecular results’ availability was 3 days (IQR: 2–5). In an area with scarce resources, the genetic characterisation supported the outbreak investigations in real time, linking cases where epidemiological investigation was limited and reassuring that the responsible strain was already circulating in Guinea. We recommend coupling thorough epidemiological and genomic investigations to control EVD clusters.

## Introduction

The Ebola virus disease (EVD) epidemic in western Africa (2013–2016), mostly affecting Guinea, Liberia and Sierra Leone, was the largest EVD epidemic ever reported [1]. The epidemic began in the area between the district of Guéckédou and Macenta in Guinea and the district of Lofa in Liberia at the end of 2013 [2-4]. By June 2016, more than 28,000 cases of EVD and more than 11,000 deaths had been reported in the three countries [5]. In Guinea, the epidemic had reached the highest transmission rates between September and December 2014, with peaks of up to 150 new confirmed cases per week. During the period April–May 2015, 9 to 28 new confirmed cases were reported per week, most of which in the prefecture of Forécariah, at the costal border with Sierra Leone, where two different lineages of Ebola virus (EBOV), namely GN1 and SL3, were simultaneously circulating [6-11].

Guinea is administratively organised into regions, which are further organised into prefectures and sub-prefectures. The prefecture of Dubréka, on the northern border of the capital city Conakry, is in the region of the ‘Basse Guinée’ or ‘Guinée Maritime’ and has a population of 328,418 inhabitants distributed in the town of Dubréka and six sub-prefectures [12].

### Outbreak detection

On 11 and 12 May 2015, the prefecture of Dubréka reported nine laboratory-confirmed cases of EVD, none of which were known contacts to the two sporadic EVD cases who had been reported in the same prefecture in the previous three weeks [13,14]. Furthermore, these new cases were spread in three villages in the same sub-prefecture and could not be linked to any known chain of transmission. In order to minimise further transmission, a field investigation was launched and a World Health Organization (WHO) rapid response team was deployed to assist the Dubréka prefecture Health Authority (*Direction Préfectorale de la Santé,* DPS). The investigation was also assisted by the diagnostic and sequencing facility of the European Mobile Laboratory (EMLab) located within the Ebola treatment centre (ETC) of Coyah. The sequencing unit of the EMLab was set up in Coyah in May 2015 as the field laboratory to perform real-time viral genome sequencing from laboratory-confirmed patients in Guinea, in order to support and guide field investigations [11]. 

Our aim is to describe the field investigation, the support provided by the real-time virus genetic characterisation, and the control measures that were put in place by the outbreak control team.

## Methods

The study was conducted within the Dubréka DPS, Guinea, in May – June 2015. The DPS of Dubréka investigated the EVD cases, supervised contact-tracing activities, and carried out active surveillance in healthcare facilities. A WHO rapid response team supported these activities. DPS community health workers followed-up the contacts of EVD cases collecting information daily on their health status. Additionally, with the support of the National Coordination, local authorities and community representatives, a campaign was organised and put in place, including door-to-door temperature screening of the population and awareness activities. Two villages were also quarantined.

### Case definitions

For the field investigation of the EVD cases in Dubréka prefecture we used the Guinea EVD definitions of confirmed, suspect, and probable cases [13]. A confirmed case was an individual testing positive for EBOV; a probable case was a deceased individual who had not been tested for EBOV and had an epidemiological link with a confirmed or a probable case. An epidemiological link was defined as a link with a confirmed or probable case or provenience from a community, which had experienced EVD cases in the previous three weeks and the staff in charge for the control of the EVD epidemic had reasonable grounds for suspecting EVD. We considered confirmed and probable cases part of the chain of transmission of Dubréka if epidemiological or phylogenetic links could be established with the cluster of cases identified on 11 and 12 May. We defined the primary case of the transmission chain as the earliest individual linked to the chain of transmission of Dubréka who developed the disease in the prefecture, but acquired the infection elsewhere.

National and international staff daily updated DPS registers of EVD cases and contacts. We used those registers, Coyah ETC patient registers, and investigation reports to describe the chains of transmission.

We described cases spatially by sub-prefecture and place of residence when a case developed symptoms suggestive of EVD [15] and temporally by International Organization for Standardization (ISO) week of first symptom onset. We used a transmission tree to visualise the transmission dynamics among cases.

We calculated (Excel 2013 and STATA 13) mean and standard deviations (SD) for numeric variables and proportions for categorical variables, using the total number of non-missing values as the denominator.

### Laboratory investigation

Virus inactivation and RNA extraction from buccal swabs, blood, or amniotic fluid samples were done using the QiAmp Viral RNA Mini Kit (Qiagen, Hilden, Germany) and EBOV was detected by real-time reverse transcription PCR (RT)-PCR with the ReaIStar Ebola virus RT-PCR Kit 1.0 (Altona, Germany) on Rotor gene (Qiagen) or Smart Cycler (Cepheid GmbH, Germany) platform, as previously described [11]. Sequencing was performed using the MinION (Oxford Nanopore Technologies, Oxford, UK) and sequences made available as part of a previous study [11]. Phylogeny was assessed by constructing a maximum likelihood phylogenetic tree under the general time-reversible (GTR) + Gamma model using RAxML [11]. The phylogenetic reconstructions were communicated to the Ebola Response National Coordination and to the Dubréka DPS, and field epidemiologists in the form of PDF and excel spreadsheet reports.

### Ethics

The National Committee of Ethics in Medical Research of Guinea (permit N°11/CNERS/14) approved the use of diagnostic residual samples and corresponding patient data for research purposes. Informed consent was not obtained from patients, as samples and patients’ information had been collected as part of the public health response to the outbreak. Epidemiological data were anonymised for the analysis.

## Results

### Epidemiological investigation

**Figure 1:**
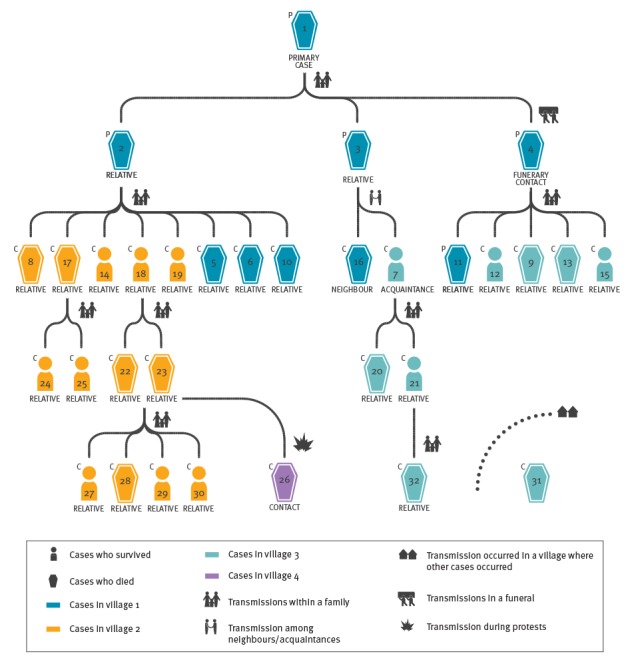
Chains of transmission among Ebola virus disease cases, Dubréka, Guinea, April–July 2015 (n = 32 cases)

All nine confirmed cases notified on 11 and 12 May represented the second generation of a newly identified chain of transmission, and were epidemiologically linked to five previously unidentified probable cases who occurred in Dubréka prefecture (Figure 1).

C: confirmed case; P: probable case.

Lines between cases represent most probable transmission links. The cases were numbered based on date of symptom onset (for case ‘11’ we used the date of outcome since the time of symptom onset was not available).

### Description of the chain of transmission of Dubréka

The case who was believed to be the primary case (case 1) was a young person who acquired the infection in a hospital outside the Dubréka prefecture during hospitalisation for conditions unrelated to EVD. By the time their roommate was diagnosed with EVD, the primary case had already returned home to a village, in the prefecture of Dubréka and fell ill. The primary case was cared for by two family members (case 2 and 3). Upon the death of the primary case, on week 16, 2015, a person from the same village (case 4) prepared the corpse for the funerary rituals. Subsequently, cases 2, 3 and 4 developed EVD compatible symptoms and died without being tested or subsequently buried by personnel trained to handle dead bodies according to the WHO standards aimed to prevent further community infections [16].

**Figure 2:**
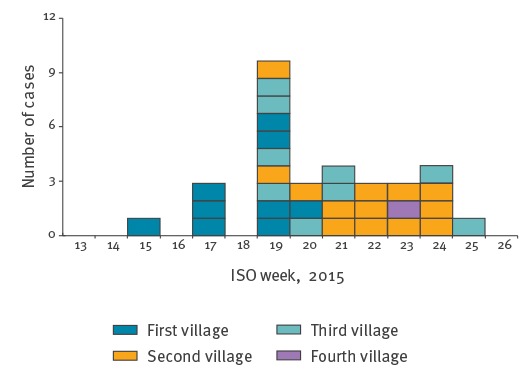
Distribution of Ebola virus disease cases by date of symptom onset^a^, prefecture of Dubréka, Guinea, April–July 2015 (n = 32 cases)

After having taken care of case 1, case 2 developed the disease and transmitted the infection to eight relatives in two villages (cases 5, 6, 8, 10, 14, 17, 18, and 19): one of them (case 17) died in the community while the others were isolated in an ETC (Figure 2).

ISO:**International Organization for Standardization.

^a ^For one case we used the date of outcome since the symptom onset was not available.

Of the seven relatives who were isolated, four died. Case 17 transmitted the infection to two family members (cases 24 and 25) who were both isolated and recovered. Case 18 transmitted the infection to two family members as well (case 22 and 23). On its way to an ETC, the ambulance transporting cases 22 and 23 was attacked and set on fire by a mob of protesters. In the aftermath of this event, case 26 developed the disease and died in the community. Subsequently, cases 22 and 23 were isolated in an ETC where they died. Four family members acquired the infection (cases 27–30) following contact with cases 22 and 23, and were isolated. One of them died. 

Case 3 transmitted the infection to two neighbours/acquaintances (cases 16 and 7), one of whom died in an ETC. The other transmitted the infection to a family member who died without being isolated (case 20), and to an additional family member who was pregnant (case 21) and, after recovering, delivered an 8 month-old stillborn (case 32) in an ETC who tested positive for EVD.

After preparing the corpse of the believed primary case for the funerary rituals, case 4 developed EVD and transmitted the infection to five family members: four (cases 9, 12, 13 and 15) were isolated and two of them died. The fifth relative (case 11) died in the community without being tested. Investigations would only reveal scarce information about this case.

Case 31 lived in one of the villages affected by the Dubréka chain of transmission. The investigators did not find any link with other known cases. After identification, the patient was isolated and died.

### Epidemiological summary

Overall, 32 cases were found, with 31 who could be linked to a single transmission chain including the primary case and four additional generations of cases (three cases in the first generation, 15 in the second, six in the third, and six in the fourth). The number of cases peaked in week 19 (4–10 May), when 10 individuals developed EVD-related symptoms; the chain of transmission ended on week 30, 2015, 21 days after the last case was discharged from an ETC. The 32 cases occurred among four villages. A total of 27 cases were confirmed, 26 acquired the infection from a relative, 24 were isolated in an ETC, and 20 died, among whom eight without being isolated. The mean and median age of the cases was 30 years (range: 3–60 years, stillbirth case excluded), seven cases (stillbirth case excluded) were younger than 18 years. Of the 32 cases, 19 were female. Overall, 994 contacts (mean = 31/case) of the cases in this chain had been listed in the contact tracing register. Since most of the cases lived in few villages and belonged to few families, 271 individuals had been listed two or more times as contacts for different cases. Among the 24 cases admitted to an ETC and with available information, the mean time between symptom onset and ETC admission was 3.2 days (SD: 2.2). Among the eight probable cases, who died in the community without being tested, the mean time between onset and death was 5.5 days (SD: 2.9).

### Virological investigation

At the time of the Dubréka outbreak, viruses circulating in Guinea were derived from two lineages: one derived from lineage GN1, referring to viruses from early cases in Guinea, and the other derived from lineage SL3, corresponding to viruses first seen in Sierra-Leone and later in Conakry at the end of 2014 [11].

**Figure 3:**
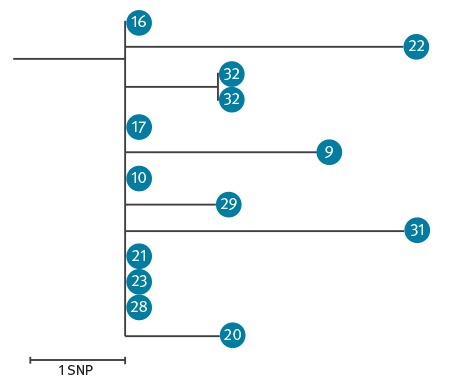
Phylogenetic tree of sequences derived from Ebola virus disease cases, Dubréka, Guinea, April–July 2015 (n = 12 cases)

We sequenced a total of 13 EBOV-positive samples from 12 patients (cases 9, 10, 16, 17, 20, 21, 22, 23, 28, 29, 31 and 32; Figure 3).

SNP: single nucleotide polymorphism.

All sequences in the phylogenetic tree belong to the SL3 lineage (data not shown). Phylogenetic reconstruction was performed by maximum likelihood under the GTR + Gamma model using RAxML as described elsewhere [11]. The numbering of the 12 samples in the blue circles, is identical to that of the case numbering used in Figure 1. Samples 32 correspond to the amniotic fluid of the mother (case 21) and the stillborn baby (case 32). The length of the branches corresponds to the number of SNPs in the respective strain(s) that distinguish them from the ancestral virus. European Nucleotide Archive accession numbers of the sequences depicted in the phylogenetic tree are as follow: LT630519; LT630531; LT630546; LT630545; LT630523; LT630516; LT630514; LT630543; LT630548; LT630528; LT630530; LT630542; LT630526.

The sequences obtained were all closely related to each other (i.e. differed by 1 to 3 single nt polymorphisms, SNPs), if not identical, and formed one cluster belonging to the SL3 lineage [11]. The order of magnitude of SNPs observed in this study is in line with the mean EBOV evolutionary rate reported by Holmes et al. during the 2013–2016 outbreak (1.20 x 10^–3^ per site per year (5% Bayesian credible interval: 1.13–1.27 x 10^–3^), which approximately corresponds to 1.89 nt changes per month (5% Bayesian credible interval: 1.78–2.00) [17]. We identified case 31 as part of the Dubréka cluster although no clear epidemiological link could be established by the investigation teams. We also demonstrated that the virus isolated from case 32 (stillborn child) was identical to the virus in the amniotic fluid (Figure 3) and had one SNP difference as compared with the blood sample of his mother (case 21).

Of the 13 EBOV-positive samples, nine were isolated from blood, three from swabs (cases 17, 20, 32) and one from amniotic fluid (case 32). The time interval between the sampling date and the release of sequencing data to response teams had a median of 3 days (IQR: 2–5) with a range of 2 to 43 days; longer time intervals corresponded to retrospective analysis of samples.

### Control measures

The response was coordinated at a local level by the Dubréka DPS and included the activities of surveillance, case management and investigation, community engagement, social mobilisation, and safe and dignified burials according to the WHO standards that included the use of personal protective equipment, use of body bags, and sanitisation of family’s environment [16]. In order to break the chain of transmission rapidly, additional control measures were implemented with the involvement of the National Coordination, district and local authorities, community members and leaders, and communities. Between 7 and 13 June, in the sub-prefecture where most of the cases originated, community health workers led by WHO supervisors performed an active door-to-door awareness campaign and temperature screening of the population. Cases of fever were further investigated. During the campaign, one confirmed case was identified and isolated. Between 1 and 10 July, two villages where a number of contacts were being followed-up were quarantined; residents were not allowed to leave the area and people were not allowed to enter. Food and primary healthcare were provided for free in the quarantined villages.

## Discussion

The chain of transmission of Dubréka contributes to explain the complexity of the west African Ebola epidemic. The chain was active for more than 3 months between April and July 2015, affecting 32 individuals in four villages of the same prefecture. The long duration was associated with a several factors, including a lack of trust toward local authorities and international organisations, the high mobility of contacts, fears of stigmatisation, lack of adequate healthcare facilities, and a limited implementation of preventive measures, especially at the onset of the outbreak. The sequencing of the viruses from confirmed cases was very useful in supporting epidemiological investigations. Genomic surveillance allowed matching of cases with transmission chains, excluding the possibility of new virus introductions during the Dubréka outbreak, and improving the timeliness of investigation. Timely field-based genomic surveillance through Nanopore technology made this possible [11]. For all cases for which it was performed, genome sequencing confirmed the findings of the epidemiological investigations and reassured investigators about case 31 for whom it was not possible to identify a chain of transmission. Genome sequencing was also essential to confirm that case 32 (the case in a stillbirth) had not only been infected with EVD, but that the strain infecting the baby was highly related to the one infecting the mother, thereby confirming transplacental transmission of EBOV.

The believed primary case acquired the infection during a hospital admission unrelated to EVD in another prefecture. EVD transmission in hospital settings is common and in some previous EVD outbreaks, hospitals acted as epidemic amplifiers [18]. Most cases acquired the infection from a relative. In addition to transmission within families and nosocomial transmission, transmission in the context of a funeral was identified in this study, consistent with what has been reported in previous EVD outbreaks [19,20].

The routine contact tracing activities carried out during the outbreak investigation were intensive and on average more than 30 contacts per case were followed-up, surpassing the WHO target of at least 10 contacts per case [21]. However, outbreak investigation activities were hindered and threatened by acts of community resistance and violence against the outbreak response team, including the wounding of two district health officials and the destruction of one ambulance. Community resistance can partially explain the high proportion of community deaths and the long time span between symptom onset and isolation of cases in the chain of transmission of Dubréka, on average 3 days vs the recommended less than 2 days [21].

In Dubréka, during the campaign of active search of cases, only one confirmed case, a known contact, was identified, while no new cases were recognised during the quarantine of two villages, suggesting that the traditional surveillance activities were on the right track. The outbreak response probably benefited from the intensified activities of community sensitisation during the campaign of active search of cases and during quarantine. Furthermore, the presence in the field of local, regional and national leaders showed a political commitment and increased the perception of risk in the community.

Our investigation suffered from some limitations. First, we cannot exclude that one or more EVD cases were missed during the investigation; however, no new cases were reported in Dubréka after July 2015. Under-ascertainment of EVD cases was an enormous challenge during the whole EVD western Africa outbreak. Second, differences in cases’ spelling of names and related information were identified between the databases kept at local, ETC, and national levels. This has been an issue identified in all of Guinea and not only in Dubréka. We reviewed the records of the field investigations to clarify inconsistencies and made sure these differences did not affect the quality of the study. The Guinea ring vaccination cluster-randomised trial to test efficacy of a new Ebola vaccine was ongoing in Dubréka during this outbreak [22,23]. The trial had a blind-design and the Dubréka DPS was not informed of which cases were vaccinated. The trial might have had an effect in interrupting part of the transmission chains, but we are not able to quantify this effect.

## Conclusions

The rapid investigation of the initially identified EVD cluster and the thorough surveillance activities performed in the prefecture of Dubréka for more than 3 months were essential to the stop transmission. Virus genomic characterisation supported the epidemiological investigation by: (i) confirming and corroborating epidemiological investigations; (ii) reassuring that all cases were associated with a single EBOV lineage (SL3), already circulating in Guinea, and therefore no novel virus introduction had occurred; (iii) linking case 31, for whom epidemiological information was limited, to the ongoing chains of transmission; (iv) confirming trans-placental transmission of EBOV. This investigation shows the feasibility and the utility of genetic characterisation in supporting EVD field outbreak investigations in settings characterised by scarcity of resources. These are characteristics that make this approach particularly suitable for the investigation in situations of new flare-ups, such as the ones that challenged surveillance systems in Guinea, Liberia, and Sierra Leone following the end of the outbreak.
